# Seroprevalence of Herpes Simplex Virus Types 1 and 2 among Pregnant Women in South-Western Romania

**DOI:** 10.3390/life14050596

**Published:** 2024-05-07

**Authors:** Cristiana Luiza Radoi, Oana Mariana Cristea, Dan Dumitru Vulcanescu, Adela Voinescu, Tiberiu Liviu Dragomir, Laurentiu Vasile Sima, Sonia Tanasescu, Octavia Oana Harich, Andrei Theodor Balasoiu, Dominic Gabriel Iliescu, Ovidiu Zlatian

**Affiliations:** 1 Doctoral School, University of Medicine and Pharmacy of Craiova, 200349 Craiova, Romania; luizacristianaradoi@gmail.com; 2 Microbiology Department, University of Medicine and Pharmacy of Craiova, 200349 Craiova, Romania; ioneteoana@yahoo.com (O.M.C.); ovidiu.zlatian@umfcv.ro (O.Z.); 3 Department of Microbiology, “Victor Babes” University of Medicine and Pharmacy, Eftimie Murgu Square 2, 300041 Timisoara, Romania; dan.vulcanescu@umft.ro (D.D.V.); adela.voinescu@umft.ro (A.V.); 4 Multidisciplinary Research Center on Antimicrobial Resistance (MULTI-REZ), Microbiology Department, “Victor Babes” University of Medicine and Pharmacy, Eftimie Murgu Square 2, 300041 Timisoara, Romania; 5 Medical Semiology II Discipline, Internal Medicine Department, “Victor Babes” University of Medicine and Pharmacy, Eftimie Murgu Sq. No. 2, 300041 Timisoara, Romania; dragomir.tiberiu@umft.ro; 6 Surgical Semiology Department, “Victor Babes” University of Medicine and Pharmacy, 300041 Timisoara, Romania; 7 Department of Pediatrics, “Victor Babes” University of Medicine and Pharmacy, Eftimie Murgu Sq. No. 2, 300041 Timisoara, Romania; tanasescu.sonia@umft.ro; 8 Department of Functional Sciences, Immuno-Physiology and Biotechnologies Center, “Victor Babes” University of Medicine and Pharmacy, No. 2 Eftimie Murgu Square, 300041 Timisoara, Romania; harich.octavia@umft.ro; 9 Department of Ophthalmology, University of Medicine and Pharmacy of Craiova, 200349 Craiova, Romania; andrei_theo@yahoo.com; 10 Department of Obstetrics and Gynecology, University of Medicine and Pharmacy of Craiova, 200349 Craiova, Romania; dominic.iliescu@yahoo.com

**Keywords:** herpes, pregnancy, Romania, seroprevalence

## Abstract

Background: Pregnancy-related infections with the human herpes simplex virus (HSV) strains HSV-1 and HSV-2 are particularly noteworthy. There are numerous reported examples of intrapartum transmission of herpes infection, notwithstanding the extreme rarity of intrauterine transfer from mother to fetus. The purpose of this study was to evaluate the seroprevalence of HSV-1 and HSV-2 antibodies in pregnant women in the western region of Romania. Methods: Pregnant women who presented for routine pregnancy monitoring at Romania’s County Clinical Emergency Hospital in Craiova between 2013 and 2016 and 2019 and 2022 were included in the study. In order to find anti-HSV-1/2 IgG antibodies, we conducted serological testing on the patients and gathered demographic information from them. Results: HSV-1 seroprevalence was shown to have declined in rural areas and increased in urban areas, with values between 2013 and 2016 being 89.30% and those between 2019 and 2022 being 84.96%, respectively. Women over 35 who were pregnant had the highest seroprevalence. The seroprevalence of HSV-2 decreased from 16.16% in 2013–2016 to 12.43% in 2019–2022, and both rural and urban areas continued to experience this declining trend. Similarly, pregnant women over 35 years old had the highest frequency of HSV-1 infections. Conclusions: Establishing educational programs and other actions to reduce the transmission rate and ultimately the prevalence of the disease can be made easier with knowledge about the seroprevalence of HSV-1 and HSV-2 infections.

## 1. Introduction

Human herpes simplex virus (HSV) is a DNA virus belonging to the Herpesviridae family and comprises two distinct species—HSV-1 and HSV-2. Traditionally, HSV-1 is associated with oral herpes, while HSV-2 is linked to genital herpes. However, in recent decades, there has been a notable shift in the etiology of the virus, with HSV-1 being increasingly implicated in approximately 30% of genital herpes cases and vice versa [1,2,3]. Notably, HSV is a significant component of the TORCH (Toxoplasma, Rubella, Cytomegalovirus, Herpes) complex, a group of pathogens known to potentially cause congenital infections during pregnancy. This evolving understanding underscores the dynamic nature of HSV infections and highlights the need for continued research to elucidate the changing landscape of herpes simplex virus epidemiology.

Primary infection with herpes simplex virus typically occurs during childhood and is symptomatic in approximately 10–25% of cases, while the majority of infections are either subclinical or asymptomatic. Following the initial infection, the virus establishes latency in the sensory nerve ganglia. Reactivation of the latent virus can be triggered by various factors, both external (exogenous) and internal (endogenous), leading to viral replication and the potential onset of clinical disease. This reactivation process underscores the dynamic nature of herpes simplex virus infections, where periods of latency can be interrupted by triggers, resulting in recurrent episodes of symptomatic disease. Understanding the factors influencing viral reactivation is crucial in managing and preventing the clinical manifestations of herpes simplex virus infections [2,4].

Neonates born to mothers with primary genital herpes can develop a herpes infection through contamination during labor, when they pass through the birth canal, or after birth through contact with the mother or other infected people. In general, neonatal herpes develops symptomatically (in the presence of maternal systemic protective antibodies). If there is a condition with poor immunity, neonatal herpes manifests as a severe generalized infection with neurological involvement and unfavorable prognosis. Children who survive are left with central nervous system sequelae. Transmission of HSV type 2 in utero via the placenta can lead to malformations in newborns (microcephaly, intracranial calcifications, and chorioretinitis) [4,5].

### Epidemiology of Herpes Virus Infections

Because of the ease of transmission, infections with HSV-1 are ubiquitous; a study from 2014 described seroprevalences of 50–70% in the general populations of industrialized nations, while it approaches ubiquity at 100% in low-income countries. Conversely, the same study reported a seroprevalence of HSV-2 with a more variable distribution, ranging from 10% to 40% in the general population, which can escalate to be between 60% and 95% in populations with heightened vulnerability, such as people living with HIV [3].

In 2020, James et al. conducted a meta-analysis study which revealed that 1816.5 million women (66.1%) had an oral HSV-1 infection, 92.5 million women (5.1%) had a genital HSV-1 infection, and 313.5 million (17.1%) women aged 15–49 years had an HSV-2 infection (Table 1) [6]. Between 1999 and 2010, the United States had a seroprevalence of anti-HSV IgG antibodies of 53.0% for HSV-1 and 15.7% for HSV-2 [7]. Subsequently, in the years 2015–2016, a different American study found that, among individuals aged 14–49, the seroprevalence of HSV-1 was 48%, but that of HSV-2 was 12% [8]. In addition, a 21.10% HSV-2 IgG seroprevalence in pregnant women was reported in the United States [9].

The seroprevalence of herpes simplex virus type 1 (HSV-1) in Asia has been empirically reported to vary between 70% and 90% in a variety of countries [10,11]. On the other hand, there is significant variability in the seroprevalence of herpes simplex virus type 2 (HSV-2), varying between 5% and 30% [11]. According to reports, the seroprevalence of HSV-1 is 75% in Australia [12]. Seroprevalence statistics in the African environment show increased rates of HSV-1, with most investigations being corroborated by seroprevalence rates more than 80% [13]. In a similar vein, estimates of HSV-2’s seroprevalence in African communities range from 30% to 80% [13,14]. HSV-1’s seroprevalence in Latin America is shown to range from 60% to 90%, while HSV-2’s seroprevalence is more widely distributed, ranging from 10% to 40% in various countries [15].

A thorough meta-analysis carried out in 2020 in Europe found that women had a mean seroprevalence of anti-HSV-1 IgG antibodies of 69.5%. Additionally, a regional breakdown of these data revealed a seroprevalence of 61.6% in the general population of Western Europe, 57.7% in Northern Europe, 77.2% in Southern Europe, and 78.7% in Eastern Europe [16]. The mean European seroprevalence of HSV-2 in women was 12.2%. The seroprevalence in the general population was determined to be 9.6% in Eastern Europe, 12.2% in Southern Europe, 13.2% in Western Europe, and 13.5% in Northern Europe following a regional breakdown [17].

**Table 1:** Seroprevalence of anti-HSV antibodies across the globe.

	Anti-HSV-2 Antibodies		Anti-HSV-2 Antibodies	
	Childbearing-Age Women	Pregnant Women	Childbearing Age Women	Pregnant Women
North America *United States*	53.0% [7] 48.0% [8]		15.7% [7] 12.0% [8]	21.10% [9]
Asia	70–90% [11]		5–30% [11]	
Australia	75.0% [12]			
Africa	>80% [13]		30–80% [13,14]	
Latin America	60–90% [15]		10–40% [15]	
Europe	69.5%		12.2% [17]	
*Eastern Europe*	78.7%		9.6%	
*Southern Europe*	77.2%		12.2%	
*Western Europe*	61.6%		13.2%	
*Northern Europe*	57.7%		13.5%	

This study aimed to examine the change in the seroprevalence of HSV infections in south-west Romania between the two time periods, providing data that can help policymakers and healthcare providers to identify at-risk groups, better allocate resources, and develop targeted prevention and control strategies.

## 2. Materials and Methods

### 2.1. Study Design and Participants

Pregnant women from south-west Romania who presented consecutively in two time periods (2013–2016—Group 1 and 2019–2022—Group 2) for routine TORCH screening at the County Clinical Emergency Hospital of Craiova, Romania, were tested for HSV-1 and HSV-2. The pregnant women were residents in the south-west region of Romania. The study design was cross-sectional and observational in nature. Demographic variables collected from the patients included age, place of residence, and area of residence.

Laboratory tests were carried out for HSV infection. Tests for anti-HSV IgG antibodies were performed during the first trimester of pregnancy. We used the 2013–2016 electrochemiluminescence method (ECLIA) (Cobas E601 analyzer, Roche Diagnostics, Basel, Switzerland). As we upgraded our analyzer to the new improved version, we used the immune chemiluminescence method for the 2019–2022 period (analyzer Architect i1000, Abbott Laboratories, Abbott Park, IL, USA) with improved sensitivity and specificity. Reagents were provided by the manufacturers. The results were classified as positive or negative for HSV infection, as instructed by the reagent package insert. 

This study was approved by the Committee of Ethics and Academic and Scientific Deontology of Craiova, Romania (approval no. 84/16.09.2020). Access to the database for this study was approved by the Ethics Committee of Clinical County Emergency Hospital of Craiova, Romania.

### 2.2. Statistical Analysis

The data were retrieved from “HIPOCRATE” hospital software and imported into STATA 17 statistical software (StataCorp Ltd., College Station, TX, USA). The numerical data were presented as mean (standard deviation), while count data were presented as count (percentage). To evaluate the differences between Groups 1 and 2, we used the chi-square test with a significance level of *p* < 0.05. Since age was normally distributed in both groups, we used the Student’s *t*-test to assess age differences between Group 1 and Group 2.

## 3. Results

### 3.1. Tested Subjects

Group 1 (355 pregnant women) was tested between 2013 and 2016, while Group 2 (1350 pregnant women) was tested between 2019 and 2022. The majority of participants in both Group 1 and Group 2 were from urban areas (59.72% and 54.96%, respectively). Additionally, the study found that the participants in Group 1 were one year younger than those in Group 2 (*p* = 0.002). The mean age of Group 1 was 28.06 ± 6.02 years, while the mean age of Group 2 was 29.15 ± 5.72 years (Table 2). In Group 1, the highest percentage of tested women was between the ages of 26 and 30 (33.80%), and in Group 2 most tested women were also in the 26–30 years age group 33.03%).

### 3.2. Seroprevalence of HSV-1 Infection

The total HSV-1 IgG seroprevalence statistically significantly decreased from 89.30% between 2013 and 2016 to 84.96% between 2019 and 2022 (*p* < 0.001), especially in urban areas, where the decrease was from 89.62% to 82.88% (Table 3).

In Group 1, the highest seroprevalence of HSV-1 IgG was in the >35 years age group (93.02%), while the lowest seroprevalence was observed in the <20 years age group. In Group 2, the highest seroprevalence was observed in women between 21 and 25 years old (87.07%), while the lowest was in women aged 26–30 years (83.86%). 

In pregnant women aged >35 years, we observed a decrease in seroprevalence (93.02% in Group 1 vs. 85.48% in Group 2). This trend of decreasing seroprevalence was maintained in all age groups, except for those under 20 years of age, where we noticed a slight increase (from 85.42% to 85.71%). None of these differences were statistically significant (*p* > 0.05).

### 3.3. Seroprevalence of HSV-2 Infection

The total prevalence of antibodies against HSV-2 decreased slightly (*p* = 0.063), from 16.16% in the 2013–2016 group to 12.43% in the 2019–2020 group. Regarding the area of residence, we noted the seroprevalence slightly decreased both in rural (from 13.70% to 11.82%) and especially in urban areas (17.84% to 12.92%) between the two time periods.

In both Group 1 and Group 2, the highest seroprevalence of HSV-2 IgG was observed in the >35 years pregnant women (21.43% and 21.92%, respectively). Conversely, the lowest seroprevalence was in the 31–35 years age group in pregnant women in Group 1 (12.33%), and in young pregnant women <20 years old in Group 2 (6.06%) (Table 4). 

In pregnant women aged 26–30 years, we observed a marked decrease in seroprevalence between the two time periods (from 18.70% to 9.68%, *p* < 0.001), which was maintained in the <20 years age group (from 16.33% to 6.06%, *p* = 0.044).

### 3.4. Epidemiology of Anti-HSV IgG Antibodies in the Dolj County

Positive anti-HSV-1 and HSV-2 IgG antibodies in pregnant women were compared with negative controls using a contingency table (Table 5). No link could be established using the following values: OR = 1.03 in Group 1 (*p* = 0.949) and OR = 0.94 in Group 2 (*p* = 0.796).

**Figure 1:**
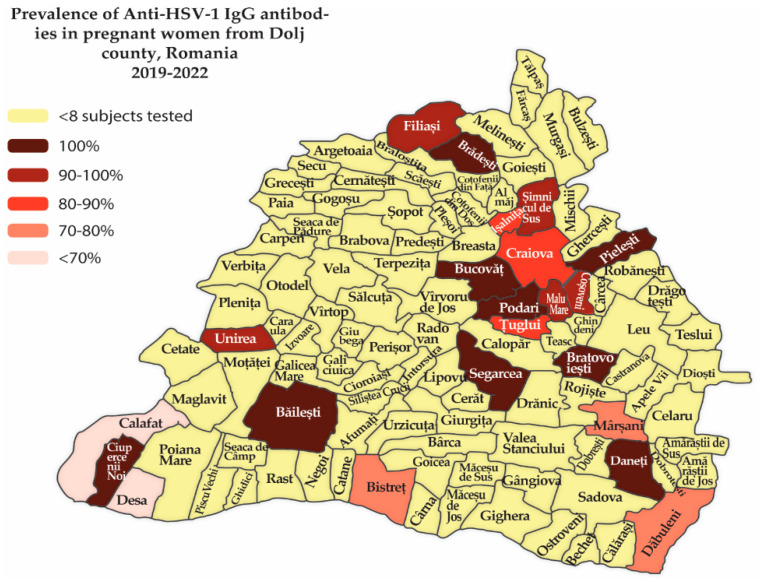
The spread of anti-HSV-1 IgG seroprevalence in pregnant women across Dolj County, Romania 2019–2022.

For the more recent cases (Group 2), most of the towns in Dolj County showed a high seroprevalence, suggesting high levels of exposure to HSV-1 in this area (Figure 1). The biggest city in the county, Craiova, had a rather high seroprevalence of 86.67%; however, there are small towns like Podari, Bailești, Bucovat, Segarcea, Ciupercenii Noi, Pielești, Bradesti și Bratovoesti that had a seroprevalence of 100%. 

**Figure 2:**
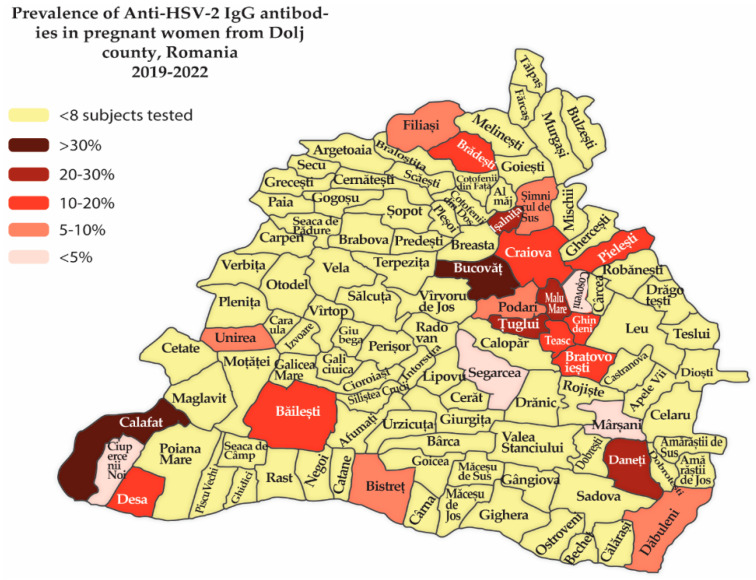
Spread of anti-HSV-2 IgG seroprevalence in pregnant women across Dolj County, Romania 2019–2022.

In regard to HSV-2 exposure, the highest prevalence percentages were recorded in the localities of Bucovat and Calafat, with values of approximately 30%. On the other hand, there were localities such as Segarcea, Cosoveni, Ciupercenii Noi, and Marsani where the percentage was 0% (Figure 2).

## 4. Discussion

In our study, there was a marked increase in the number of tests carried out between 2013–2016 and 2019–2022, as reported by other researchers from Romania who studied various agents in the TORCH complex [18,19,20]. Most pregnant women were from rural areas, as shown by studies of pregnant women performed in 2015 and 2016 [21,22]. The increase in mean age by one year in Group 2 can be attributed to the development of in vitro fertilization that allows women to become pregnant at a higher age and to other factors that cause young women to delay conceiving a child until they reach social and financial stability in the development of Romanian society [23]. 

Our findings showed a seroprevalence of anti-HSV-1 of 89.30% between 2013 and 2016 and of 84.96% between 2019 and 2022. A study performed in the USA reported a seroprevalence of 59.3% among women aged 20–39 years [9]. A meta-analysis performed in Australia reported a pooled mean seroprevalence of 79.70%. Another meta-analysis of European studies found a pooled seroprevalence of 67.40% [16]. We observed that the seroprevalence HSV-1 was high in our region compared with the rest of the world. Regarding the decline in seroprevalence observed in our study, the same meta-analysis found a decrease of 1% per year in the pooled seroprevalence. This is consistent with our findings (89.30% between 2013 and 2016 and 84.96% between 2019 and 2022), with a decrease of −4.34% between the two periods separated by three years.

In Romania, we could find only two studies reporting HSV seroprevalence: a study published in 2008 reported an 87.30% seroprevalence in pregnant women in the country capital, Bucharest [24], and another study from 2010 reported an 88.00% seroprevalence in the same area in women aged 15–49 years [25].

Most of the pregnant women in our study were aged between 21 and 35 years. In women aged 26–30 years, the seroprevalence was 88.33% between 2013 and 2016 and 83.86% between 2019 and 2022. In comparison, a study published in 2018 on 1215 pregnant women from the USA found lower seroprevalences of 58.50% in the 20–29 age group and 60.50% in the 30–39 age group [9].

The higher seroprevalence of anti-HSV-1 IgG reported by us in urban areas could be explained by the higher population density, which leads to more frequent social interpersonal contact through kissing, hugging, handshaking, or sharing foods and drinks [12]. Regarding the age distribution of HSV-1 seroprevalence, the higher prevalence observed in the 21–25 years age group could be due to the higher probability of young women living in urban areas as part of the common migration for school or work. The higher prevalence in women aged >35 years may be due to increased exposure to HSV-1 early in life, and for biological reasons such as a decline in immunity with age, and it is well known that there is an increased prevalence of herpetic infections in immunodeficient patients [26,27,28]. 

The decrease in the seroprevalence of anti-HSV-1 antibodies observed from 2013–2016 to 2019–2022 may be due to an increased awareness of the virus and its transmission. The female population may also have increased their use of personal protective anti-infectious measures and hygiene practices, particularly after the COVID-19 pandemic [29,30].

Similarly, we observed a decrease in the seroprevalence of anti-HSV-2 IgG antibodies from 16.16% in the 2013–2016 group to 12.43% in the 2019–2022 group. The reasons are common with those enumerated for HSV-1 seroprevalence decrease [2], with the added importance of decreased sexual transmission in the context of improving sexual education, protection, and sexual health in general [31,32,33].

Our study found a total seroprevalence of 16.16% between 2013 and 2016 and 12.43% between 2019 and 2022. These figures are consistent with the results of a meta-analysis performed in 2015 on 111 studies reporting seroprevalence of anti-HSV-2 IgG antibodies in various populations around the globe [34], which showed a global seroprevalence between 10.2% and 21.8% in females aged between 15 and 49 years. Regionally, the seroprevalence was 5.2–17.8% and 13.7–24.6% in the Americas, 30–47.1% in Africa, and 4.8–14.8% in South East Asia. In Romania, a seroprevalence of 15.10% was reported in a study published in 2008, which was comparable to our results [24].

In our study, the seroprevalence in the <20 years age group of pregnant women decreased markedly (from 16.33% to 6.06%), as did the seroprevalence in the 26–30 years age group (from 18.70% to 9.68%). A study performed in 2018 on pregnant women from the USA reported a seroprevalence of 16.8% in the 20–29 age group and of 27.8% in the 30–39 years age group [9]. In another study performed in Romania in 2010 [24], the seroprevalence was 11.00% in women aged 15–19 years, and 38.30% in the 40–44 years age group. The higher seroprevalence in women aged >35 years obtained in our study (21.43%/21.92%) can be explained by the presumed higher number of sexual contacts with different partners and the natural increase in the risk of acquiring the infection as women age [8]. In addition, older women have increased knowledge about sexual health and use preconception measures more frequently [35]. The seroprevalence rates observed in older age groups suggest the need to target younger populations for prevention efforts. To achieve this, educational initiatives should be implemented in schools to raise awareness about the modes of transmission of herpetic infections, preventive measures, and the importance of early testing. By educating younger people before they are exposed to risk factors, we can change the trajectory of seroprevalence in future generations. Additionally, increasing access to healthcare for younger people ensures that they can receive preventive services, including vaccinations, routine screenings, and counseling on safe practices.

The Dolj county is located in south-west Romania, and the high seroprevalence of herpes simplex infections could be related to cultural, social, or lifestyle aspects. The fact that the county has a southern border with Bulgaria can impact seroprevalence because the trans-border migration of people can impact the spread of the virus. 

We can observe that all towns around Craiova had a high seroprevalence of herpes simplex infections, but this probably reflects easier access to testing rather than a genuinely high seroprevalence, because in Dolj County, the testing center in Craiova is the only state-supported TORCH testing center in the region. Consistent with most studies [2,3,12], the big towns in the county like Filiași, Băilești and Segarcea have the highest seroprevalence. In big cities, there is also increased population mobility, which can lead to a dilution of seroprevalence, compared with smaller closed communities.

It is noteworthy that the difference in seroprevalence of anti-HSV-2 IgG between the big city of Craiova and small towns in the county suggests better access to testing, better health education, and improved sexual protection.

## 5. Conclusions

This is the largest study performed in Romania on HSV infection prevalence in pregnant women. The results showed a high seroprevalence of HSV-1 in pregnant women from south-west Romania that decreased between the two time periods, above the levels found in the European and global data, confirming the descending trend. Regarding HSV-2 infection, the prevalence was again higher in many European countries and showed a descending trend. 

Knowing the seroprevalence of HSV-1 and HSV-2 infections can help in establishing educational programs and other interventions to lower the transmission rate and eventually the prevalence of the disease. However, we must keep in mind that prepartum transmission of HSV from mother to child is extremely rare, and most of the time the infection is transmitted during birth. Therefore, they can be easily prevented by performing cesarean section instead of birth. The mother’s HSV serology is not informative for these reasons, and most countries do not recommend HSV screening as a routine for pregnant women.

One possible policy intervention, considering the seroprevalence observed, is to implement routine screening for the herpes simplex virus (HSV) in all pregnant women during their first prenatal visit. This approach is essential for the early detection of HSV infections, which is critical for preventing transmission to newborns.

Potential avenues for future research could explore the root causes of the observed variations in seroprevalence or assess the efficacy of specific public health policies. Moreover, potential methodological advancements exist for future seroprevalence studies, such as longitudinal designs to better track seroprevalence changes over time and correlate them with additional demographic variables beyond age and area of residence, in order to better target health interventions. Utilizing media platforms that cater to diverse demographics, particularly women of reproductive age who increasingly rely on these platforms for healthcare education, can effectively disseminate public health messages. Tailoring content that resonates with the lifestyles and perceptions of younger individuals can enhance the impact of health communication strategies.

## Figures and Tables

**Table 2:** Demographic characteristics of the pregnant women tested for IgG anti-HSV-1/2 antibodies.

	Group 1 (2013–2016) *N* = 355	Group 2 (2019–2022) *N* = 1350	*p* Value
Area of residence (n/%)			
Rural	143 (40.28%)	608 (45.04%)	0.108
Urban	212 (59.72%)	742 (54.96%)	
Age (years) Mean (standard deviation)	28.06 (6.02)	29.15 (5.72)	0.002 *
Age range (years)	15–44	14–47	

*: *p* < 0.05, statistically significant.

**Table 3:** Comparative seroprevalence of anti-HSV-1 IgG antibodies between the two groups, stratified by area of residence and age group.

Variable	Group 1 (2013–2016) *N* = 355	Group 2 (2019–2022) *N* = 1350	*p* Value
**Total prevalence**	317/355 (89.30%)	1147/1350 (84.96%)	<0.001 *
**Area of residence**			
Rural	127/143 (88.81%)	532/608 (87.50%)	0.667
Urban	190/212 (89.62%)	615/742 (82.88%)	0.017 *
**Age group**			
<20 years	41/48 (85.42%)	84/98 (85.71%)	0.962
21–25 years	65/72 (90.28%)	229/263 (87.07%)	0.462
26–30 years	106/120 (88.33%)	374/446 (83.86%)	0.225
31–35 years	65/72 (90.28%)	301/357 (84.31%)	0.192
>35 years	40/43 (93.02%)	159/186 (85.48%)	0.187
**Area of residence and age group**			
RURAL, <20 years	31/36 (86.11%)	64/75 (85.33%)	0.913
RURAL, 21–25 years	39/42 (92.86%)	149/170 (87.65%)	0.340
RURAL, 26–30 years	34/39 (87.18%)	173/194 (89.17%)	0.718
RURAL, 31–35 years	14/16 (87.50%)	100/114 (87.72%)	0.980
RURAL, >35 years	9/10 (90.00%)	46/55 (83.64%)	0.608
URBAN, <20 years	10/12 (83.33%)	20/23 (86.96%)	0.771
URBAN, 21–25 years	26/30 (86.67%)	80/93 (86.02%)	0.929
URBAN, 26–30 years	72/81(88.89%)	201/252 (79.76%)	0.063
URBAN, 31–35 years	51/56 (91.07%)	201/243 (82.72%)	0.121
URBAN, >35 years	31/33 (93.94%)	113/131 (86.26%)	0.228

*: *p* < 0.05, statistically significant.

**Table 4:** Comparative seroprevalence of anti-HSV-2 IgG antibodies between the two groups, stratified by area of residence and age group.

Variable	Group 1 (2013–2016) *N* = 355	Group 2 (2019–2022) *N* = 1350	*p* Value
**Total prevalence**	58/355 (16.16%)	168/1350 (12.43%)	0.063
**Area of residence**			
Rural	20/142 (13.70%)	72/607 (11.82%)	0.533
Urban	38/213 (17.84%)	96/743 (12.92%)	0.068
**Age group**			
<20 years	8/49 (16.33%)	6/99 (6.06%)	0.044 *
21–25 years	9/72 (12.50%)	34/267 (12.73%)	0.958
26–30 years	23/119 (18.70%)	43/438 (9.68%)	<0.001 *
31–35 years	9/73 (12.33%)	44/355 (12.39%)	0.988
>35 years	9/42 (21.43%)	41/187 (21.92%)	0.970
**Area of residence and age group**			
RURAL, <20 years	3/37 (8.11%)	5/75 (6.67%)	0.781
RURAL, 21–25 years	6/42 (14.29%)	24/173 (13.87%)	0.944
RURAL, 26–30 years	5/40 (12.50%)	20/193 (10.36%)	0.691
RURAL, 31–35 years	3/17 (17.65%)	12/113 (10.62%)	0.398
RURAL, >35 years	3/10 (30.00%)	11/55 (20.00%)	0.479
URBAN, <20 years	5/12 (41.67%)	1/24 (4.17%)	0.004 *
URBAN, 21–25 years	3/30 (10.00%)	10/94 (10.64%)	0.921
URBAN, 26–30 years	18/79 (21.69%)	23/249 (9.16%)	0.003 *
URBAN, 31–35 years	6/56 (10.71%)	32/242 (13.22%)	0.612
URBAN, >35 years	6/32 (18.75%)	30/132 (22.73%)	0.626

*: *p* < 0.05, statistically significant.

**Table 5:** Prevalence of Anti-HSV-1 IgG and anti-HSV-2 IgG antibodies.

	Group 1 (2013–2016) *N* = 355			Group 2 (2019–2022) *N* = 1350		
	Anti-HSV-1 IgG (+)	Anti-HSV-1 IgG (−)	Total	Anti-HSV-1 IgG (+)	Anti-HSV-1 IgG (−)	Total
Anti-HSV-2 IgG (+)	51 (16.19%)	6 (15.79%)	57 (16.15%)	149 (12.35%)	26 (13.00%)	167 (12.44%)
Anti-HSV-2 IgG (−)	266 (83.81%)	32 (84.21%)	296 (83.85)	1001 (87.65%)	174 (87.00%)	1175 (87.56%)
Total	317 (100%)	38 (100%)	355 (100%)	1150 (100%)	200 (100%)	1350 (100%)
Odds	0.193	0.188	0.193	0.141	0.149	0.142
Odds ratio	1.03			0.94		
95% CI	0.397–3.170			0.596–1.539		
*p* value	0.949			0.796		

## Data Availability

The data presented in this study are available upon request from the corresponding author.
